# Plasma complement factor H is associated with disease activity of patients with ANCA-associated vasculitis

**DOI:** 10.1186/s13075-015-0656-8

**Published:** 2015-05-21

**Authors:** Su-Fang Chen, Feng-Mei Wang, Zhi-Ying Li, Feng Yu, Ming-Hui Zhao, Min Chen

**Affiliations:** Renal Division, Department of Medicine, Peking University First Hospital, Peking University Institute of Nephrology, Beijing, 100034 China; Key Laboratory of Renal Disease, Ministry of Health of China, Beijing, 100034 China; Key Laboratory of Chronic Kidney Disease Prevention and Treatment, Peking University, Ministry of Education, Beijing, 100034 China; Peking-Tsinghua Center for Life Sciences, Beijing, 100034 China

## Abstract

**Introduction:**

Increasing evidences have demonstrated that activation of alternative complement pathway plays an important role in the pathogenesis of anti-neutrophil cytoplasmic antibody (ANCA)-associated vasculitis (AAV). The current study aimed to investigate the association of complement factor H (CFH), a key regulator of the alternative complement pathway, with the disease activity of AAV.

**Methods:**

Plasma CFH levels were measured in 82 patients with myeloperoxidase (MPO)-AAV in active stage. Of the 82 patients, plasma CFH levels of 27 patients were longitudinally measured. Serum anti-CFH autoantibodies were screened in AAV patients. Circulating complement activation profiles including C4d, Bb, C3a, C5a and soluble C5b-9 of AAV patients in active stage were further detected. Associations between plasma CFH levels and clinicopathological parameters as well as the prognosis were analyzed.

**Results:**

Plasma CFH levels were significantly lower in active AAV patients compared with AAV patients in remission and normal controls. Correlation analysis showed that plasma CFH levels inversely correlated with initial serum creatinine, Birmingham Vasculitis Activity Score (BVAS), proportion of total crescents and cellular crescents in renal specimens, and circulating levels of C3a, C5a and Sc5b-9, meanwhile positively correlated with estimated glomerular filtration rate (eGFR), hemoglobin levels and circulating levels of C3. Moreover, multivariate survival analysis revealed that plasma CFH levels were independently associated with composite outcome of death or end stage renal disease (ESRD) in AAV patients, after adjusting for age, gender, hemoglobin level and urinary protein (P = 0.03, HR 0.85, 95 % CI 0.73–0.98) or adjusting for age, gender, total crescents (%) and urinary protein (P = 0.03, HR 0.85, 95 % CI 0.73–0.98), while not as an independent predictor after adjusting for age, gender, serum creatinine and urinary protein (P = 0.57, HR 0.96, 95 % CI 0.83–1.11).

**Conclusion:**

In conclusion, plasma CFH levels are associated with disease activity, and, to some extent, associated with composite outcomes of patients with MPO-ANCA-associated vasculitis.

## Introduction

Anti-neutrophil cytoplasmic antibody (ANCA)-associated vasculitis (AAV) is a group of systemic autoimmune diseases, characterized by pauci-immune necrotizing small-vessel vasculitis and circulating autoantibodies against neutrophil cytoplasmic constituents, especially proteinase 3 (PR3) and myeloperoxidase (MPO). AAV comprises granulomatosis with polyangiitis (GPA), microscopic polyangiitis (MPA), eosinophilic granulomatosis with polyangiits (EGPA), and renal-limited vasculitis (RLV) [1].

The complement system, composed of more than 30 plasma-bound and membrane-bound proteins, plays a central role in innate and humoral immunity, and can be triggered for three different pathways: the classical pathway, the lectin pathway, or the alternative pathway. Recently, increasing evidence from both animal studies and clinical studies has demonstrated that activation of alternative complement pathway plays a critical role in the pathogenesis of AAV [2–8]. In particular, levels of Bb, which reflect activation of the alternative pathway, in renal histology, urine, and circulation, are associated with the disease activity of AAV [7, 8].

Actually, there are many soluble and membrane-bound complement regulators protecting cells and tissues from unintended injury by the complement cascade. Complement factor H (CFH), an abundant 155-kDa soluble glycoprotein composed of 20 short consensus repeats (SCR), is a key regulator of the alternative complement pathway in the circulation and on cell surfaces. CFH competes with factor B for binding to C3b, thus impeding formation of alternative-pathway C3 convertases (C3bBb), accelerates the decay of the alternative pathway C3-convertase (C3bBb), and acts as a cofactor for the factor I-mediated proteolytic inactivation of C3b [9]. The cofactor/decay accelerating activity is mediated by SCR1–SCR4 in the N-terminus, while SCR19 and SCR20 in the C-terminus are essential for cell surface regulation of CFH [10]. In addition, CFH contains specific binding sites for C-reactive protein (CRP) [11]. Abnormalities of CFH resulting in dysregulation of the alternative complement pathway have been involved in the pathogenesis of several autoimmune diseases, including atypical hemolytic uremic syndrome (aHUS) and C3 nephropathy [12, 13]. However, the association of CFH and AAV is far from clear. In the current study, we detected plasma levels of CFH in AAV patients in both active stage and remission, and the associations between plasma CFH levels and clinicopathological characteristics as well as prognosis were further analyzed.

## Methods

### Patients and blood samples

Eighty-seven patients with active AAV with complete clinical and pathological data diagnosed in the Department of Nephrology, Peking University First Hospital from 2007 to 2013 were initially enrolled in this study. However, five patients with PR3-ANCA-positive vasculitis were excluded because increasing evidence suggests that MPO-AAV and PR3-AAV are two distinct entities [14, 15]. Therefore, all of the remaining 82 patients were positive for perinuclear ANCA (P-ANCA) and MPO-ANCA at diagnosis. All these patients met the criteria of the 2012 Chapel Hill Consensus Conference definition for AAV [1]. Patients with secondary vasculitis or with any other coexisting renal disease, such as anti-glomerular basement membrane nephritis, lupus nephritis, IgA nephropathy, diabetic nephropathy, and membranous nephropathy, were excluded. Plasma samples from these patients were collected on the day of renal biopsy and before the initiation of immunosuppressive therapy. Of the 82 patients with AAV, plasma samples from 27 patients who achieved complete remission defined as described previously [16] were also collected at their regular ambulatory visits.

Plasma and serum samples of 65 age-matched and gender-matched healthy blood donors and 30 patients with renal biopsy-proven lupus nephritis in the active stage were collected as normal controls and disease controls, respectively. All patients with lupus nephritis fulfilled the 1997 American College of Rheumatology revised criteria for systemic lupus erythematosus (SLE) [17].

The blood samples of all participants were centrifuged at 2000 × *g* for 15 minutes at 4 °C within 30 minutes after collection. The plasma was then stored in aliquots at −80 °C until use. Repeated freeze/thaw cycles were avoided. Informed consent was obtained from each participant. The research was in compliance with the Declaration of Helsinki and was approved by the ethics committees of Peking University First Hospital.

### Clinical evaluation

The vasculitis disease activity was measured by the Birmingham Vasculitis Activity Score (BVAS) [18]. Remission was defined as “absence of disease activity attributable to active disease qualified by the need for ongoing stable maintenance immunosuppressive therapy” (complete remission), or “at least 50 % reduction of disease activity score and absence of new manifestations” (partial remission), as described previously. Treatment resistance was defined as unchanged or increased disease activity in patients with acute AAV after 4 weeks of treatment with standard therapy or a reduction of <50 % in the disease activity score after 6 weeks of treatment, or chronic persistent disease defined as the presence of at least one major item or three minor items on the disease activity score list after >12 weeks of treatment [16].

The renal response to treatment, evaluated at 6 months after initiation of immunosuppressive therapy, was judged according to the following criteria, which were described previously [19–21]: (1) complete recovery of renal function was indicated by normalization of renal function and resolution of hematuria; (2) partial recovery of renal function was indicated by stabilization or improvement of renal function, with serum creatinine ≥133 μmol/l but dialysis independent; and (3) treatment failure was indicated by progressive decline in kidney function with persistence of active urinary sediment despite immunosuppressive therapy.

The patients were followed up in outpatient clinics specified for AAV. The primary end point was defined as death and the secondary end point was defined as end stage renal disease (ESRD). The composite end point was defined as death or ESRD.

### Quantification of plasma CFH

The detection of plasma CFH was according to the methods described previously [22, 23], with minor modification. In brief, serial concentrations of commercially available highly purified human factor H from 1050 to 16.4 μg/ml were used to develop the standard curve. The CFH level of each sample was calculated using Curve expert 1.3 (Hyams DG, Starkville, MS, USA). The linear portion of the standard curve was subsequently used for the measurement of plasma CFH. All assays were run in duplicate, and when standard errors were over 10 %, samples were routinely re-analyzed.

### Detection of serum anti-CFH autoantibodies using ELISA

Serum anti-CFH autoantibodies were detected by enzyme-linked immunosorbent assay (ELISA), slightly modified as described previously [24]. In brief, the 96-well plate was coated with commercial highly purified human factor H in 4 μg/ml phosphate-buffered saline (PBS) or PBS alone as antigen-free wells. After blocking with 1 % bovine serum albumin (BSA), samples diluted 1:50 in PBS containing 0.1 % Tween-20 (PBST) were added to both antigen-coated and antigen-free wells and followed by incubation with alkaline phosphatase-conjugated goat anti-human IgG. Color developed with substrate solution was measured using an ELISA reader at 405 nm.

### Quantification of circulating C3, C4d, Bb, C3a, C5a, and soluble C5b-9 levels

Serum C3 was detected at the day of renal biopsy using a rate nephelometry assay (IMMAGE, normal range >0.85 g/l; Beckman-Coulter, Brea, California, USA). The measurement of plasma concentrations of human complement components, including complement fragments C4d, Bb, C3a, C5a, and soluble C5b-9, were performed using commercial ELISA kits according to the manufacturer’s instructions (Quidel, San Diego, CA, USA).

### Renal histopathology

The renal biopsy specimens were routinely examined by light microscopy, direct immunofluorescence, and electron microscopy. Biopsies were separately scored by two pathologists blinded to the clinical data, according to the previously standardized protocol for scoring renal biopsies of patients with AAV [25–27]. The presence of glomerular lesions, including crescents (cellular/fibrous), glomerulosclerosis (local/segmental/global), and fibrinoid necrosis, as well as a number of other lesions, were calculated as the percentage of the total number of glomeruli in a biopsy. Interstitial and tubular lesions were scored semiquantitatively on the basis of the percentage of the tubulointerstitial compartment that was affected: tubular atrophy (– for 0 %; + for 0–50 %, ++ for >50 %), interstitial infiltrates (– for 0 %, + for 0–20 %, ++ for 20–50 %, +++ for >50 %), and interstitial fibrosis (– for 0 %, + for 0–50 %, ++ for >50 %).

### Statistical analysis

Quantitative data were expressed as mean ± standard deviation (for data that were normally distributed), or median and interquartile range (for data that were not normally distributed). Comparison of normally distributed quantitative parameters between two groups was assessed using the *t* test. In the case of multiple comparisons, one-way analysis of variance (ANOVA) followed by Bonferroni multiple-comparison test was performed. Comparison of normally distributed paired variables was assessed using the paired-samples *t* test. Differences of qualitative results were compared using the chi-square test. Pearson’s test or Spearman’s test was used for correlation analysis as appropriate. Kaplan–Meier curves were performed to analyze the outcomes using the log-rank test. All variables presented in Table 1 were assessed using univariate survival analysis. If the *P* value was <0.05, this predictor was allowed to be included in multivariable Cox regression models. Age and sex were forced into multivariable models, because they were potential confounding factors according to previous studies [28, 29]. Because of the close correlation between initial serum creatinine and hemoglobin (Hb) (rho = 0.56, *P* <0.001), initial serum creatinine and total crescents (%) (rho = 0.55, *P* <0.001), these parameters were separately included in the multivariate analysis using Models A, B and C. Results were expressed as hazard ratio (HR) with 95 % confidence interval (CI). Receiver operating characteristic (ROC) curve analysis was performed to assess the accuracy of plasma CFH level for distinguishing patients with active disease from remission, and prognosis prediction. The optimal cutoff values were constructed according to the ROC curves. Analysis was performed with SPSS statistical software package (SPSS11.0; SPSS Inc., Chicago, IL, USA). Differences were considered significant if *P* <0.05.

**Table 1:** Clinical and histopathological parameters of patients with active AAV

Parameter	Value
Number of patients	82
Age (mean ± SD)	60.3 ± 13.0
Gender (male/female)	41/41
MPA/GPA	68/14
Initial serum creatinine (μmol/l) (median (IQR))	321.0 (176.8, 637.3)
eGFR (ml/minute/1.73 m^2^)^a^ (median (IQR))	13.86 (6.51, 27.85)
Dialysis dependent at presentation	30 (36.6 %)
Urinary protein (g/24 hours) (mean ± SD)	1.95 ± 1.56
Skin rash	10 (12.2 %)
Arthralgia	26 (31.7 %)
Muscle pain	19 (23.2 %)
Pulmonary involvement	56 (68.3 %)
Ear, nose, and throat involvement	38 (46.3 %)
Ophthalmic involvement	16 (19.5 %)
Gastrointestinal involvement	17 (20.7 %)
Nervous system involvement	15 (18.3 %)
BVAS (mean ± SD)	21.1 ± 5.6
Hemoglobin (g/dl) (mean ± SD)	9.1 ± 1.9
ESR (mm/1 hour) (mean ± SD)	77.2 ± 39.9
Glomerular lesions (%)	
Total crescents (mean ± SD)	54.1 ± 28.3
Cellular crescents (mean ± SD)	42.0 ± 26.7
Fibrous crescents (median (IQR))	3.9 (0, 15.9)
Tubulointerstitial lesions	
Tubular atrophy (−/+/++)	6/59/17
Interstitial infiltration (−/+/++/+++)	1/19/57/5
Interstitial fibrosis (−/+/++)	16/58/8

^a^eGFR (ml/minute per 1.73 m^2^) =175 × (plasma creatinine^)−1.234^ × age^−0.179^ × 0.79 (if female) [30].

*AAV* anti-neutrophil cytoplasmic antibody-associated vasculitis, *BVAS* Birmingham Vasculitis Activity Score, *eGFR* estimated glomerular filtration rate, *ESR* erythrocyte sedimentation rate, *GPA* granulomatosis with polyangiitis, *IQR* interquartile range, *MPA* microscopic polyangiitis, *SD* standard deviation

## Results

### Demographic and general data

Of the 82 patients with active AAV, 41 were male and 41 were female, with an age of 60.3 ± 13.0 (range 14–83) years at diagnosis. The general data for these patients are presented in Table 1.

### Plasma CFH levels in patients and controls

**Fig. 1:**
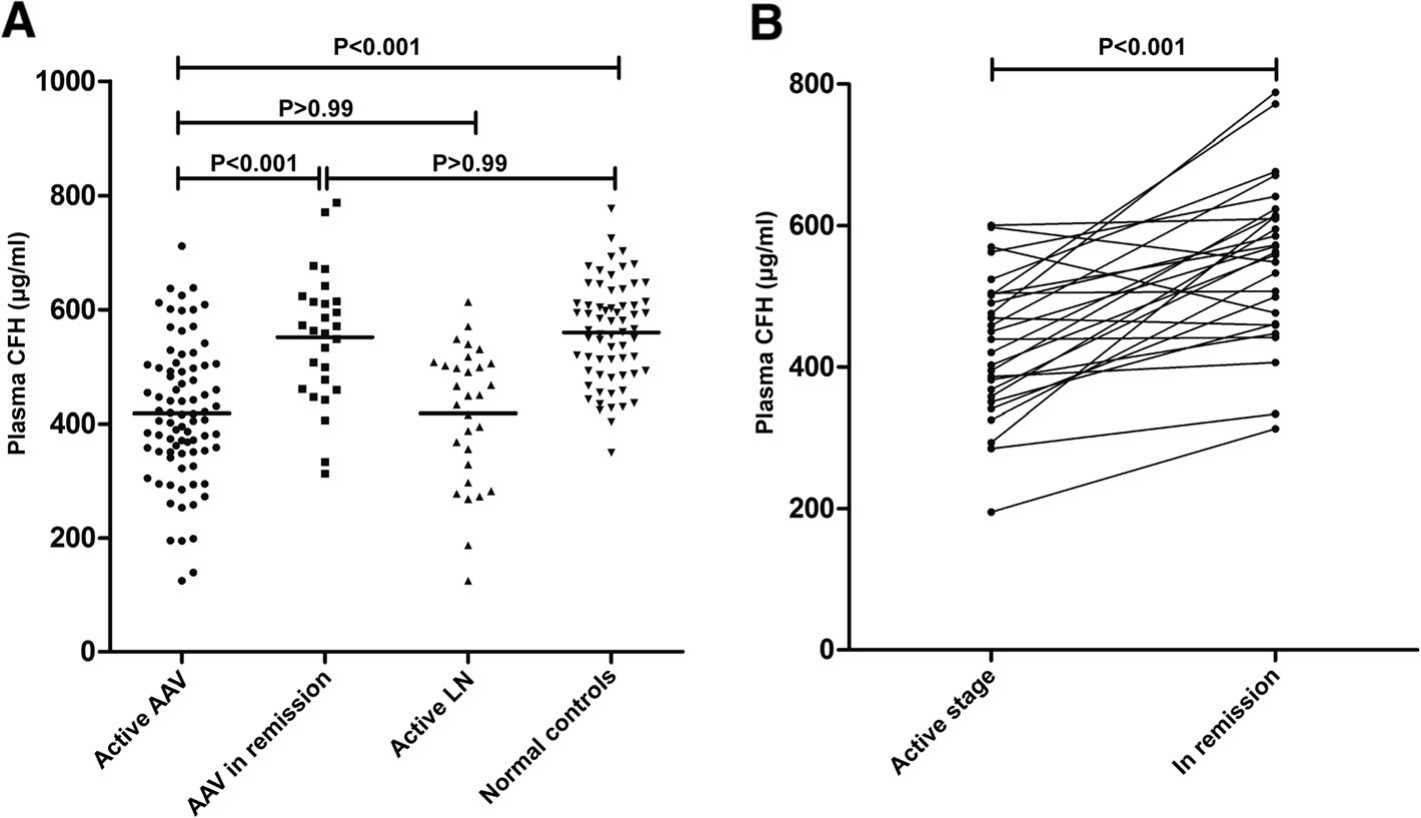
Plasma levels of complement factor H (CFH) in different groups. **a** Plasma levels of CFH in anti-neutrophil cytoplasmic autoantibody-associated vasculitis (AAV) patients in the active stage and in remission, patients with lupus nephritis (LN), and normal controls. **b** Changes of plasma CFH levels in 27 AAV patients with sequential plasma samples

Plasma CFH levels of patients with AAV in the active stage and remission, patients with lupus nephritis, and normal subjects are shown in Fig. 1a. Plasma levels of CFH were significantly lower in patients with AAV in the active stage compared with patients in remission and normal controls (417.87 ± 119.74 vs. 551.33 ± 114.12 μg/ml, *P* <0.001; 417.87 ± 119.74 vs. 559.72 ± 87.92 μg/ml, *P* <0.001, respectively). There was no significant difference in the plasma CFH levels between AAV patients in remission and normal controls (551.33 ± 114.12 vs. 559.72 ± 87.92 μg/ml, *P* >0.99). No significant difference was observed in plasma levels of CFH between active AAV and active lupus nephritis patients (417.87 ± 119.74 vs. 418.06 ± 119.51 μg/ml, *P* >0.99).

We then compared plasma levels of CFH in 27 AAV patients with plasma samples from both the active stage and remission. The plasma levels of CFH were significantly higher in remission than those in the active stage (551.33 ± 114.12 vs. 431.72 ± 100.91 μg/ml, *P* <0.001) (Fig. 1b). Twenty-five of these 27 patients had an increase in plasma level of CFH in remission compared with in the active stage, whereas only two patients had a slight decrease in plasma level of CFH in remission.

### Associations between plasma CFH levels and clinicopathological parameters of patients with active AAV

**Fig. 2:**
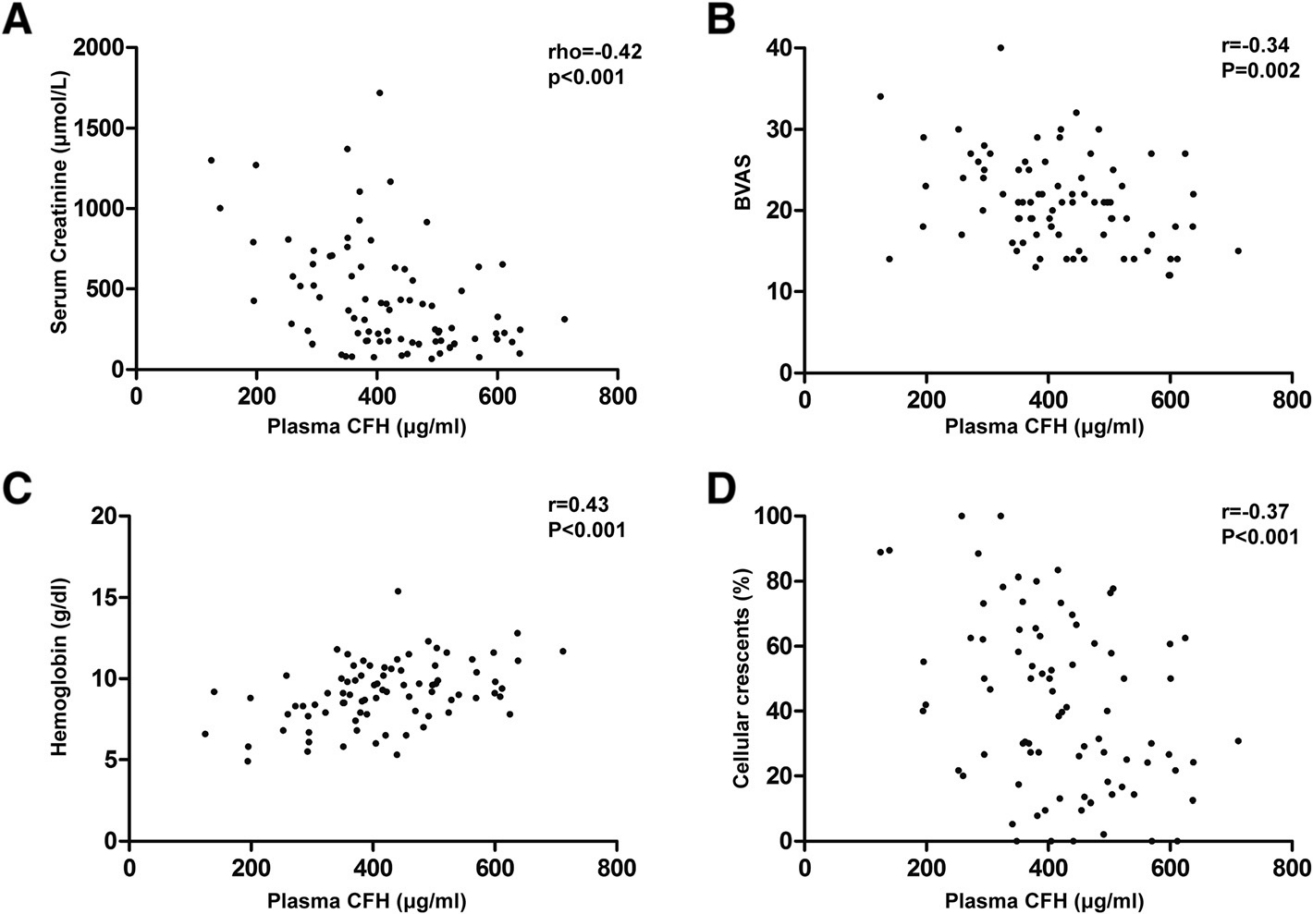
Plasma levels of complement factor H (CFH) correlated with **a** initial serum creatinine, **b** Birmingham Vasculitis Activity Score (BVAS), **c** hemoglobin, **d** proportion of cellular crescents

Associations between plasma CFH levels and clinicopathological parameters of patients with active AAV are shown in Fig. 2. Correlation analysis showed that plasma CFH levels inversely correlated with initial serum creatinine and BVAS (rho = −0.42, *P* <0.001; *r* = −0.34, *P* = 0.002, respectively), and positively correlated with estimated glomerular filtration rate (eGFR) [30] and Hb level (rho = 0.43, *P* <0.001; *r* = 0.43, *P* <0.001, respectively). For renal pathological parameters, plasma CFH levels inversely correlated with the proportion of total crescents and cellular crescents in the renal specimen (*r* = −0.33, *P* = 0.003; *r* = −0.37, *P* <0.001, respectively).

### Associations between plasma CFH levels and circulating C3, C4d, Bb, C3a, C5a, and soluble C5b-9 levels in patients with active AAV

The circulating complement parameters of patients with active AAV are presented in Table 2. We further found that plasma CFH levels positively correlated with serum levels of C3 (*r* = 0.49, *P* <0.001) and inversely correlated with some complement activation fragments, including C3a, C5a, and Sc5b-9 (rho = −0.33, *P* = 0.02; rho = −0.41, *P* = 0.005; *r* = −0.57, *P* <0.001, respectively).

**Table 2:** Circulating complement profile of patients with active AAV

	Value	Reference value [7]
Serum C3 (mg/ml) (mean ± SD)	0.89 ± 0.27	>0.85
Plasma Bb (μg/ml) (median (IQR))	1.17 (0.70, 1.86)	0.63 ± 0.26
Plasma C3a (ng/ml) (median (IQR))	1872.10 (464.20, 2354.95)	100.87 ± 70.55
Plasma C5a (ng/ml) (median (IQR))	32.77 (10.97, 72.66)	8.19 ± 5.44
Plasma Sc5b-9 (ng/ml) (mean ± SD)	894.71 ± 357.48	360.82 ± 164.51

*AAV* anti-neutrophil cytoplasmic antibody-associated vasculitis, *IQR* inter-quartile range, *SD* standard deviation

### Association between plasma CFH levels and outcomes of AAV patients

Of the 82 active AAV patients, resistance to induction therapy occurred in seven patients (8.5 %). There was no significant difference in plasma CFH levels between patients who were resistant to induction therapy and patients who achieved remission (complete or partial) (380.31 ± 83.92 vs. 421.38 ± 122.38 μg/ml, *P* = 0.39). However, as for renal response to treatment, 16 patients (19.5 %) had renal treatment failure 6 months after the initiation of immunosuppressive therapy, and plasma CFH levels were found to be significantly lower in patients with renal treatment failure compared with those achieving recovery of renal function (complete or partial) (364.33 ± 119.17 vs. 430.85 ± 117.10 μg/ml, *P* = 0.04).

**Fig. 3:**
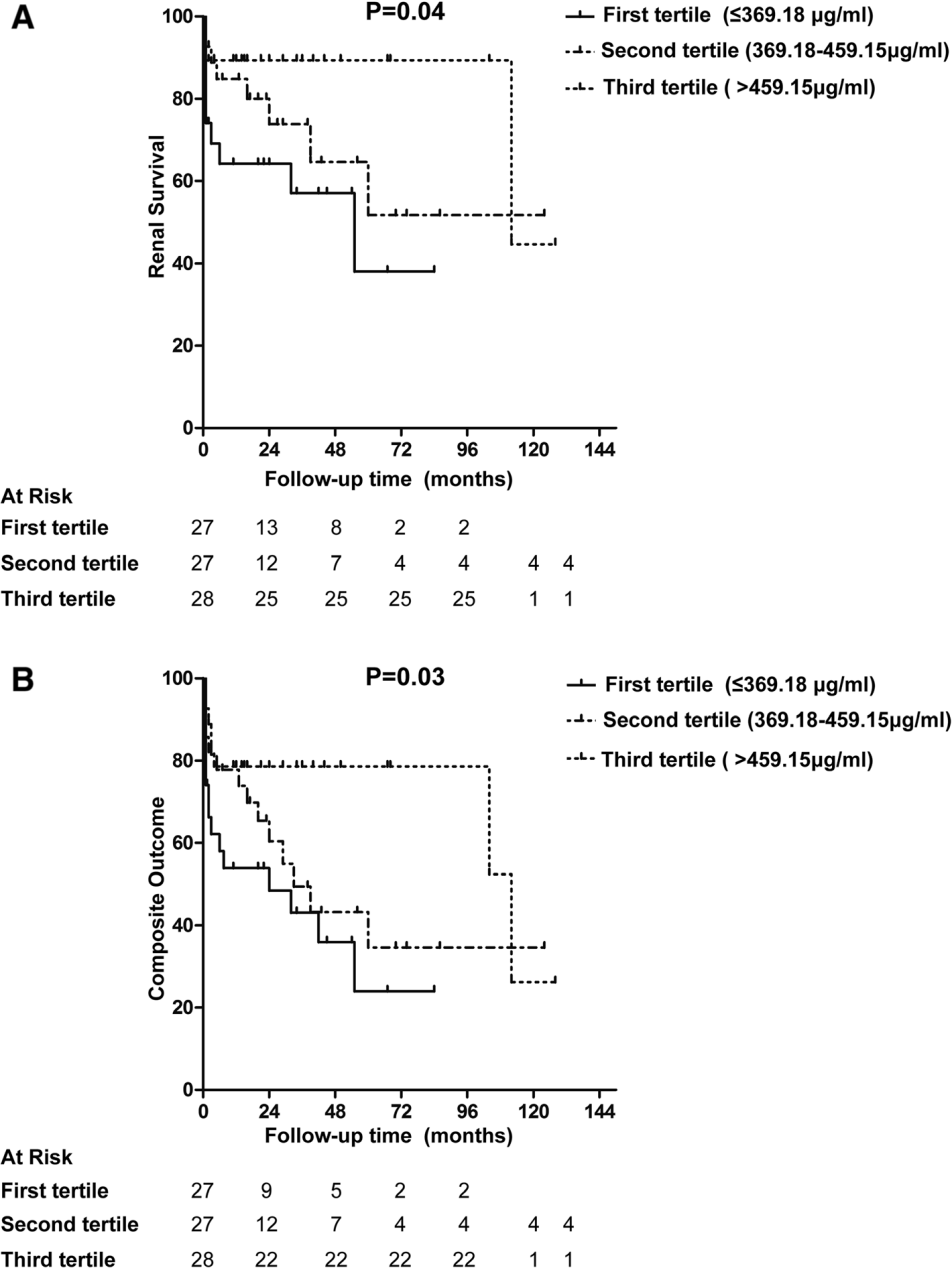
Associations between plasma levels of CFH and prognosis of patients with AAV. **a** Associations with renal survival. **b** Associations with composite outcomes (death or ESRD)

In our cohort, patients with AAV were followed up for a median period of 29 (range 1–138) months. During the follow-up, 24 patients died, 23 patients developed ESRD, and 38 patients reached the composite end point. To evaluate the association between plasma CFH levels and patients’ prognosis, patients were divided into three groups of equal size according to plasma CFH levels. Kaplan–Meier survival analysis showed that there were no significant difference in all-cause mortality among these three groups (*P* = 0.78). However, plasma CFH levels were found to be associated with renal survival and composite outcome of death or ESRD among these three groups (*P* = 0.04; *P* = 0.03, respectively) (Fig. 3); patients within the first tertile of plasma CFH levels (≤369.18 μg/ml) had the poorest renal outcome and composite outcome.

In univariate survival analysis of renal prognosis in patients with AAV, we found that plasma CFH level was a risk factor for renal outcome (*P* = 0.01, HR 0.79 (per 50 μg/ml increase), 95 % CI 0.67–0.95) and was also associated with composite outcome (*P* = 0.003, HR 0.81 (per 50 μg/ml increase), 95 % CI 0.71–0.93). Besides plasma CFH level, other candidate parameters entered into the multivariate analysis are presented in Table 3. The plasma CFH level failed to be an independent risk factor for ESRD in any of the multivariate models. However, since death acts as an early competing event for subsequent ESRD and analysis might be influenced by death censoring, the composite outcome was employed. This analysis revealed that the plasma CFH level was still an independent predictor for the composite endpoint after adjusting for age, gender, Hb level, and urinary protein in Model A (*P* = 0.03, HR 0.85, 95 % CI 0.73–0.98) or for age, gender, total crescents (%), and urinary protein in Model B (*P* = 0.03, HR 0.85, 95 % CI 0.73–0.98) (Table 3), while the plasma CFH level was not an independent predictor after adjusting for age, gender, serum creatinine, and urinary protein in Model C (*P* = 0.57, HR 0.96, 95 % CI 0.83–1.11).

**Table 3:** Multivariate analysis of composite outcome in patients with AAV

	*P* value	Hazard ratio	95 % confidence interval
Model A			
Age	0.69	0.99	0.96–1.02
Gender (male vs. female)	0.32	1.44	0.71–2.91
Hemoglobin (≤9 g/dl vs. >9 g/dl)	0.31	1.47	0.70–3.11
Urinary protein (per g/24 hour)	0.02	1.29	1.05–1.58
Plasma CFH levels (per 50 μg/ml increase)	0.03	0.85	0.73–0.98
Model B			
Age	0.72	0.99	0.96–1.03
Gender (male vs. female)	0.36	1.39	0.69–2.82
Total crescents (%) (>50 % vs. ≤50 %)	0.26	1.69	0.68–4.17
Urinary protein (per g/24 hour)	0.09	1.21	0.97–1.52
Plasma CFH levels (per 50 μg/ml increase)	0.03	0.85	0.73–0.98
Model C			
Age	0.31	1.02	0.99–1.05
Gender (male vs. female)	0.75	1.12	0.55–2.29
Initial serum creatinine (per mg/dl)	<0.001	1.24	1.12–1.36
Urinary protein (per g/24 hour)	0.002	1.34	1.12–1.62
Plasma CFH levels (per 50 μg/ml increase)	0.57	0.96	0.83–1.11

*AAV*, anti-neutrophil cytoplasmic antibody-associated vasculitis, *CFH* complement factor H

The ROC curve analysis showed that the plasma level of CFH could, to some extent, distinguish patients with active AVV from remission (area under the curve (AUC) 0.79, 95 % CI 0.70–0.89, *P* < 0.001). At the cutoff value of 441.74 μg/ml for CFH, the sensitivity was 61 % and the specificity was 89 %. The ROC curve was also plotted to verify the accuracy of CFH for composite outcome prediction. The AUC was 0.68 (95 % CI 0.56–0.80, *P* = 0.008). This value suggested an optimal cutoff of 434.57 μg/ml for plasma CFH, providing a sensitivity of 79 % and a specificity of 57 %.

### Detection of serum CFH autoantibodies in patients with AAV

Serum autoantibodies against CFH could not be detected in patients with AAV.

## Discussion

Recently, cumulating evidence has demonstrated that activation of the alternative complement pathway plays a critical role in the pathogenesis of AAV. As a key regulator of the alternative complement pathway, abnormalities of CFH have been associated with many autoimmune diseases, including aHUS, C3 nephropathy, and SLE [12, 13, 23, 31]. Moreover, association of a novel CFH mutation with severe crescentic and necrotizing glomerulonephritis was reported [32]. However, the role of CFH in AAV is still far from clear. To our best of knowledge, there is a lack of studies on circulating CFH in patients with AAV.

In our present study, we found that plasma levels of CFH were significantly lower in patients with AAV in active stage compared with patients in remission and normal controls. Further analysis showed that plasma CFH levels inversely correlated with initial serum creatinine, BVAS, and the proportion of total crescents and cellular crescents in renal specimens, but positively correlated with eGFR and Hb. These results indicated that plasma CFH levels could reflect both systemic and renal disease activity in patients with AAV. Moreover, plasma CFH levels were found to be associated with restoration of renal function (complete or partial) and, to some extent, associated with the composite outcome of AAV patients. However, when initial serum creatinine was taken into account, it failed to be an independent risk factor, which might be relevant to the close correlation between CFH and renal function. As a retrospective study, and limited by the sample size, the value of plasma CFH level as a predictor for patients’ outcomes needs further investigation.

Our present study found a close association between CFH and renal damage in patients with MPO-ANCA-positive vasculitis. Data from the MPO-AAV mouse model have confirmed that the alternative complement pathway is required for induction of necrotizing and crescentic glomerulonephritis by anti-MPO IgG [2]. Our previous study also found that circulating Bb was associated with the severity of renal injury in patients with MPO-AAV [7, 8]. CFH is a key regulator that controls the alternative complement activation at the level of C3 convertase. Our current study found that plasma CFH levels inversely correlated with circulating levels of C3 split product, C3a, and further correlated with the final common pathway activated products, C5a and Sc5b-9, which may contribute to endothelium damage through enhanced neutrophil activation or direct effects on membranes resulting in cell lysis. However, as a retrospective and observational study, it is difficult to sort out whether the decreased plasma CFH levels are related to excessive consumption because of overactivation of complement in patients with active AAV, or are an initial factor predisposing patients to AAV susceptibility by insufficiently regulating the activation of alternative complement pathway. Previous findings of aHUS suggested that some specific CFH gene mutations could lead to quantitative CFH deficiency [12]. A genetic mechanism is unlikely to be the case in AAV patients, however, since plasma CFH levels were restored during disease remission compared with active disease. The exact mechanism is of great interest for further study.

Because an overlap of plasma CFH levels between AAV patients and normal controls occurred, we speculate that the quantitative deficiency of plasma CFH may be one aspect. Previous studies have revealed that autoantibodies against CFH and genetic variations resulted in dysfunction of CFH were important mechanisms involved in the pathogenesis of aHUS [12]. However, our current study showed that serum autoantibodies against CFH could not be detected in patients with AAV, so other underlying mechanisms of CFH in AAV are worthy of further study. Interestingly, our previous work found that MPO, which can be released from ANCA-stimulated neutrophils, could inhibit the binding between modified/monomeric C-reactive protein (mCRP) and CFH, and thus might inhibit the negative regulation of alternative complement activation [33]. This work indicated another potential way in which CFH may be involved in the development of AAV, but the detailed mechanism needs further investigation.

In the current study, only patients with MPO-ANCA were included, since previous experimental and clinical studies that investigated the role of the complement system in the pathogenesis of AAV focused mainly on MPO-ANCA-positive vasculitis [2–5, 7, 8]. Moreover, Chinese patients with AAV are predominantly MPO-ANCA-positive, as demonstrated by our previous studies [34, 35]. Whether the same is true for PR3-ANCA-positive vasculitis requires further investigation.

## Conclusions

The current study found that plasma levels of CFH are associated with disease activity and, to some extent, associated with composite outcomes of patients with MPO-AAV.

## Abbreviations

AAV: Anti-neutrophil cytoplasmic antibody-associated vasculitis
aHUS: Atypical hemolytic uremic syndrome
ANCA: Anti-neutrophil cytoplasmic antibody
ANOVA: Analysis of variance
AUC: Area under the curve
BSA: Bovine serum albumin
BVAS: Birmingham Vasculitis Activity Score
CFH: Complement factor H
CI: Confidence interval
CRP: C-reactive protein
eGFR: Estimated glomerular filtration rate
EGPA: Eosinophilic granulomatosis with polyangiits
ELISA: Enzyme-linked immunosorbent assay
ESRD: End stage renal disease
GPA: Granulomatosis with polyangiitis
Hb: hemoglobin
HR: Hazard ratio
mCRP: Modified/monomeric C-reactive protein
MPA: Microscopic polyangiitis
MPO: Myeloperoxidase
P-ANCA: Perinuclear anti-neutrophil cytoplasmic antibody
PBS: Phosphate-buffered saline
PR3: Proteinase 3
RLV: Renal-limited vasculitis
ROC: Receiver operating characteristic
SCR: Short consensus repeats
SLE: Systemic lupus erythematosus
