# Erratum to: Plasma complement factor H is associated with disease activity of patients with ANCA-associated vasculitis

**DOI:** 10.1186/s13075-017-1298-9

**Published:** 2017-05-02

**Authors:** Su-Fang Chen, Feng-Mei Wang, Zhi-Ying Li, Feng Yu, Ming-Hui Zhao, Min Chen

**Affiliations:** 1 Renal Division, Department of Medicine, Peking University First Hospital, Peking University Institute of Nephrology, Beijing, 100034 China; 2 Key Laboratory of Renal Disease, Ministry of Health of China, Beijing, 100034 China; 3 Key Laboratory of Chronic Kidney Disease Prevention and Treatment, Peking University, Ministry of Education, Beijing, 100034 China; 4 Peking-Tsinghua Center for Life Sciences, Beijing, 100034 China

## Erratum

Unfortunately, after publication of this article [1], it was noticed that there was an error in the funding information located in the Acknowledgements section. The full correct Funding section can be seen below.

**Acknowledgments**

This study is supported by a grant from the Chinese 973 project (No.2012CB517702) and four grants from the National Natural Science Fund (No.81425008, No. 81321064, No. 81370829, and No. 81400724). The authors are very grateful to Prof. Xue-Ying Li for the advice on statistical analysis.

They gratefully acknowledge the contribution of the Department of Clinical Laboratory, Peking University First Hospital.

## References

[1] Chen SF, Wang FM, Li ZY, Yu F, Zhao MH, Chen M (2015). Plasma complement factor H is associated with disease activity of patients with ANCA-associated vasculitis. Arthritis Res Ther.

